# A submerged dielectric barrier discharge plasma inactivation mechanism of biofilms produced by *Escherichia coli* O157:H7, *Cronobacter sakazakii*, and *Staphylococcus aureus*

**DOI:** 10.1038/srep37072

**Published:** 2016-11-15

**Authors:** Muhammad Saiful Islam Khan, Eun-Jung Lee, Yun-Ji Kim

**Affiliations:** 1 Department of Food Biotechnology, University of Science and Technology, Daejeon, 305-350, Republic of Korea; 2 Food Safety Research Group, Korea Food Research Institute, Seongnam-si, Gyeonggi-Do, Republic of Korea

## Abstract

A submerged dielectric barrier discharge plasma reactor (underwater DBD) has been used to inactivate biofilm produced by three different food-borne pathogens, namely *Escherichia coli* O157:H7 (ATCC 438), *Cronobacter sakazakii* (ATCC 29004), and *Staphylococcus aureus* (KCCM 40050). The inactivation that were obtained after 90 minutes of plasma operation were found to measure 5.50 log CFU/coupon, 6.88 log CFU/coupon and 4.20 log CFU/coupon for *Escherichia coli* O157:H7 (ATCC 438), *Cronobacter sakazakii* (ATCC 29004), and *Staphylococcus aureus* (KCCM 40050), respectively. Secondary Electron Images (SEI) obtained from Field Emission Scanning Electron Microscopy (FE-SEM) show the biofilm morphology and its removal trend by plasma operation at different time intervals. An attenuated total reflectance Fourier transform infrared (ATR-FTIR) measurement was performed to elucidate the biochemical changes that occur on the bacterial cell and extracellular polymeric substance (EPS) of biofilm during the plasma inactivation process. The ATR-FTIR measurement shows the gradual reduction of carbohydrates, proteins, and lipid and DNA peak regions with increased plasma exposure time. The presence of an EPS layer on the upper surface of the biofilm plays a negative and significant role in its removal from stainless steel (SS) coupons.

Microorganisms are able to aggregate on wet surfaces and grow into microcolonies to form biofilms. Biofilms are microbial communities that attach to a surface, and they are embedded in a matrix composed of exopolysaccharides and excreted nucleic acids. Biofilms have major implications for a variety of industries, including the food industry, and they create a persistent source of contamination that leads to nosocomial infections, food spoilage, and damage to industrial pipelines^1^,^2^,^3^. Food spoilage and deterioration not only result in massive economic damage, but food safety is a key priority of today’s globalizing market, with its international transportation and consumption of fresh and minimally processed food stuffs^4^. Biofilm formation can cause damage through heat transfer^5^ and the corrosion of metal surfaces^6^, although these problems are not common in the food industry. The microorganisms inside the biofilms are well-protected from disinfectants, which increases their possibility of surviving and thereby increases the contamination of food. Hence, the effective inactivation of biofilms is very important.

Conventional methods of sterilizing free-living bacteria by chemical, physical or biological methods are often ineffective with biofilms because the bacteria within the biofilm exhibit different properties from those in planktonic forms^7^,^8^. The use of moist heat and pressure via autoclave is still an inexpensive method for many applications. However, it cannot be applied to all situations and to every material, such as thermosensitive materials. Chemicals such as ethylene oxide allow for low-temperature disinfection, but ethylene oxide is both mutagenic and carcinogenic. Other chemicals, such as chlorine, formaldehyde and H_2_O_2_, are also not suitable for many applications, and they pose an environmental hazard as well as risks to human health^9^. In the case of UV and gamma ray irradiation, the energetic photons that are generated can seriously damage the structure of DNA and, most importantly, these photons are carcinogenic to the human body^10^. For all the reasons discussed above, biofilm control and removal demands the development of new strategies.

Thus, a variety of research efforts are ongoing around the world to develop novel techniques that can remove biofilms effectively. Among the non-thermal inactivation techniques, increased attention has been devoted to physical inactivation methods such as the plasma inactivation technique^11^. Thermal and non-thermal atmospheric plasmas are used for biological sterilization. The use of thermal plasma is limited by its high temperature (2,000–10,000 K), which can damage tissues^12^. However, the non-thermal plasma method works at room temperature, and because of its high efficiency and safety, it has been shown to be appropriate for various applications such as the surface modification of polymers^13^,^14^,^15^, air purification^16^,^17^, and sterilization for biological and medical purposes^18^,^19^,^20^,^21^.

Underwater electrical discharge is a fast and reliable non-thermal plasma technique that has been used extensively for microbial inactivation^22^,^23^,^24^,^25^,^26^,^27^,^28^,^29^. An environmentally friendly underwater electrical discharge method has been widely studied in the context of environmental and wastewater treatment^27^,^30^,^31^, and it has recently been introduced to bacterial inactivation studies for food safety purposes^32^. A number of underwater electrical discharge techniques have been introduced to inactivate bacteria, and different reduction levels for *Escherichia coli* O157:H7 were obtained using different discharges and devices^32^,^33^. Various reactive species were reported to give rise to different inactivation levels for *Escherichia coli* O157:H7. The generation of different types of reactive species occurs because of variations in electrical discharge; for example, pressure waves and UV light are generated by pulsed high-current underwater discharge; low pH and hydrogen peroxide (H_2_O_2_) are generated by gliding arc discharge; and atomic oxygen, atomic hydrogen and hydroxyl (OH) radical species are generated by capillary discharge. Dielectric barrier discharges (DBD) produce ozone, UV light, reactive oxygen species (ROS) and reactive nitrogen species (RNS), which are highly capable of causing bacterial inactivation^12^. Additionally, variations in gas compositions can give rise to differences in the inactivation of food-borne pathogens because different gas compositions can produce different types of reactive species^34^. Air, oxygen, nitrogen, argon, and helium are commonly used as feed gases for plasma generation. Because argon and helium are expensive, both air and nitrogen may have greater potential for use in industrial applications. Although air gas has often been used to generate plasma^35^,^36^,^37^, nitrogen gas has been used equally often as air in studies of bacterial pathogen inactivation^38^,^39^,^40^. The complete elimination of microorganisms depends on several factors such as the plasma power, the gas, the type of bacteria, and the type of medium.

The sterilization mechanism of biofilm for pathogenic bacteria by underwater DBD has not been completely elucidated^41^; hence, performing a detailed composition analysis of bacterial cell and EPS is very important. However, performing a detailed analysis of EPS is difficult because EPS is often a complex mixture of proteins, carbohydrates, lipids, DNA, and humic acid substances^42^. Furthermore, even though carbohydrates have been identified as one of the major components of EPS, the biochemical properties of these compounds remain elusive because of their complex structures and unique monomer linkages^43^,^44^,^45^. However, to gain more insight into the biochemical and physiological changes that occur during the destruction of biofilms at the molecular level, an attenuated total reflectance Fourier transform infrared (ATR-FTIR) spectroscopic analysis was performed.

The examination of the optical emission spectrum showed that the dielectric barrier discharge (DBD) produced ROS and RNS along with ozone and UV light^46^. These reactive species have a microbial inactivation effect even though this method still exhibits drawbacks such as a low penetration effect. In this study, underwater electrical discharge using a DBD plasma reactor (underwater DBD), which was originally developed by Y. S. Mok and co-workers^47^,^48^, was applied to three different types of reference strains, namely *Escherichia coli* O157:H7 (ATCC 438), *Cronobacter sakazakii* (ATCC 29004), and *Staphylococcus aureus* (KCCM 40050), to understand the inactivation mechanism of these pathogens by plasma jet.

## Results

### Biofilm formation and plasma inactivation

**Figure 1:**
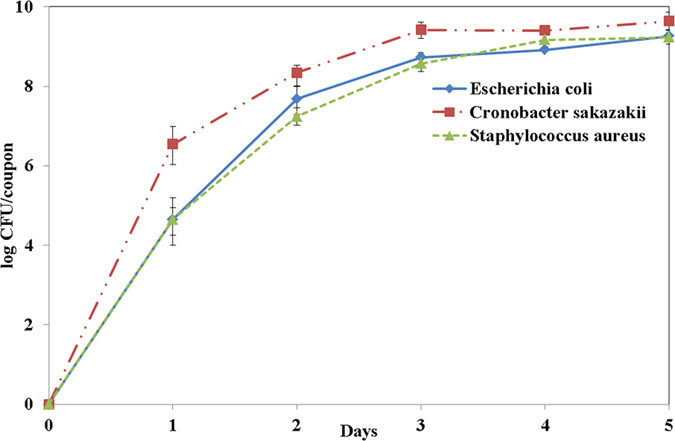
Populations of three different bacterial strains grown on the surface of stainless. Steel coupons that were immersed in TSB solution for 5 days.

Biofilms were generated on stainless steel coupons under laboratory conditions for three reference strains of the most frequently encountered food-borne pathogens, such as *Escherichia coli* O157:H7 (ATCC 438), *Cronobacter sakazakii* (ATCC 29004), and *Staphylococcus aureus* (KCCM 40050), to observe the degradation effect and inactivation mechanism of plasma on food-borne pathogens. Figure 1 shows the growth curve of pathogenic microbes on stainless steel for five days. The growth characteristics obtained over five days under laboratory conditions are quite similar for all three different types of pathogens used in this study. The populations of the three microbes were 9.26 log/coupon, 9.64 log/coupon and 9.23 log/coupon for *Escherichia coli* O157:H7 (ATCC 438), *Cronobacter sakazakii* (ATCC 29004), and *Staphylococcus aureus* (KCCM 40050), respectively.

**Figure 2:**
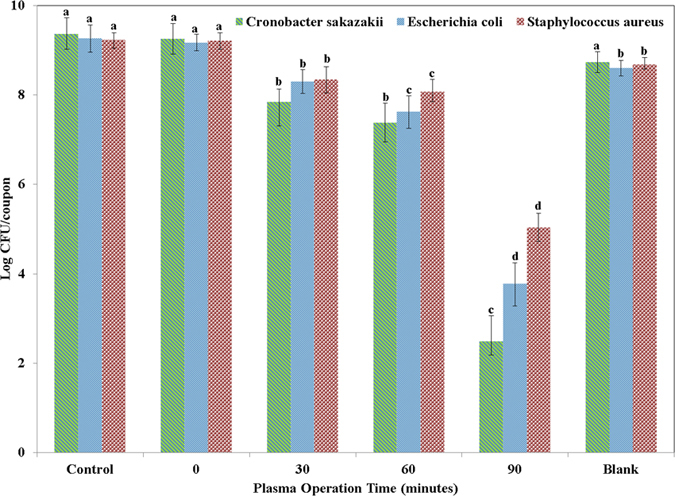
Viability assay for 5-day-old biofilm suspended in 5 ml of PBS solution; it was subjected to plasma treatment for 0, 30, 60, and 90 min, and there was a control and a blank. Treatments with different letters in the same strains were significantly different based on Duncan’s multiple ranges test (p < 0.05).

Underwater DBD plasma was employed on a stainless steel (SS) coupon containing a biofilm from the three reference strains *Escherichia coli* O157:H7 (ATCC 438), *Cronobacter sakazakii* (ATCC 29004), and *Staphylococcus aureus* (KCCM 40050). The SS coupon was placed in the acryl bath containing 4.0 L of distilled water and the submerged DBD electrodes. Clean, dry air gas was blown as a feed gas for generating plasma. The sample that was collected after one minute of airflow alone without starting the plasma discharge was considered as collected at 0 minutes, and the other samples were collected 30, 60, and 90 minutes following the start of plasma discharge. Figure 2 shows the viability of the biofilm for three different pathogenic microbes that were determined with respect to the DBD plasma treatment time, and their surviving numbers were observed in three samples in colony-forming units (CFU). The inactivation levels that were obtained after 90 minutes of plasma treatment were 5.50 log CFU/coupon, 6.88 log CFU/coupon and 4.20 log CFU/coupon for *Escherichia coli* O157:H7 (ATCC 438), *Cronobacter sakazakii* (ATCC 29004), and *Staphylococcus aureus* (KCCM 40050), respectively. *Cronobacter sakazakii* (ATCC 29004) is the most susceptible to plasma and *Staphylococcus aureus* (KCCM 40050) is the least. The majority (~90%) of the bacterial inactivation was observed after 60 minutes of plasma treatment.

A blank experiment was performed to rule out the effects of water circulation on biofilm detachment from stainless steel coupons over a 90-minute treatment, in the absence of plasma (with the plasma source turned off). Although the water circulation decreases the viable count by approximately 1 to 5%, mostly during the first minute of air flow, the inactivation difference observed for 90 minutes of water flow was much smaller than the inactivation observed in association with plasma operation (Fig. 2). This observation suggests that cell detachment occurs because of the plasma treatment, and not because of water circulation.

### Morphology study

**Figure 3:**
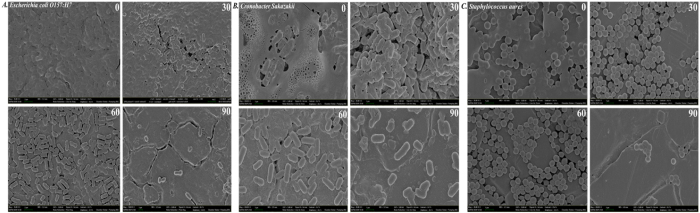
Secondary Electron Images (SEI) of 5-day-old (**A**) *Escherichia coli* O157:H7 (ATCC 43894), (**B**) *Cronobacter sakazakii* (ATCC 29004), and (**C**) *Staphylococcus aureus* (KCCM 40050) biofilm. 0, 30, 60 and 90 on the top right corner of the images represents underwater DBD treatment time.

The SE images obtained from FE-SEM show the morphology of biofilms produced by *Escherichia coli* O157:H7 (ATCC 438), *Cronobacter sakazakii* (ATCC 29004), and *Staphylococcus aureus* (KCCM 40050) at different time intervals of the plasma operation, as shown in Fig. 3. Among the three biofilms, *Cronobacter sakazakii* (ATCC 29004) produces the thickest layer (Fig. 3B-0) of EPS, which can be distinguished even by the naked eye because of its thick and sticky nature. From the morphology study, we observed that the maximum bacterial reduction occurs during the 60 to 90-minute plasma treatment; the removal/destruction of the EPS layers occurs mostly during the first 60 minutes.

### EPS characterization

**Figure 4:**
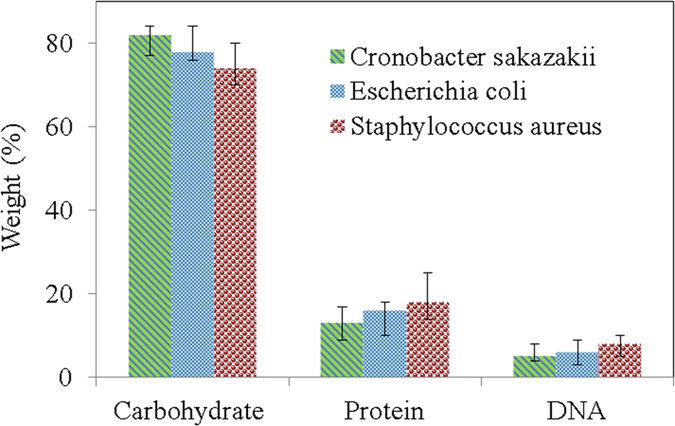
Comparison of the biochemical compositions of EPS obtained from the biofilm of *Escherichia coli* O157:H7 (ATCC 438), *Cronobacter sakazakii* (ATCC 29004), and *Staphylococcus aureus* (KCCM 40050).

To establish the proper inactivation mechanism for biofilms produced by pathogens, understanding the biochemical compositions in terms of the carbohydrates, proteins and DNA measurements of the EPS layer was imperative. The biochemical compositions of the *Escherichia coli* O157:H7 (ATCC 438), *Cronobacter sakazakii* (ATCC 29004), and *Staphylococcus aureus* (KCCM 40050) reference strains were measured, and they are plotted in Fig. 4. The EPS layers for the three different types of pathogens do not show significant differences in their biochemical compositions.

### ATR-FTIR analysis

**Figure 5:**
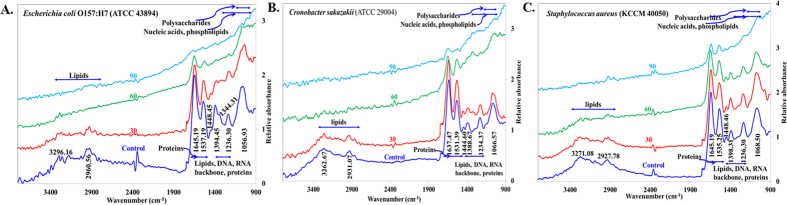
ATR-FTIR spectra of (**A**) *Escherichia coli* O157:H7 (ATCC 43894), (**B**) *Cronobacter sakazakii* (ATCC 29004), and (**C**) *Staphylococcus aureus* (KCCM 40050) biofilm for the control and after underwater DBD operations at 30, 60 and 90 min.

**Figure 6:**
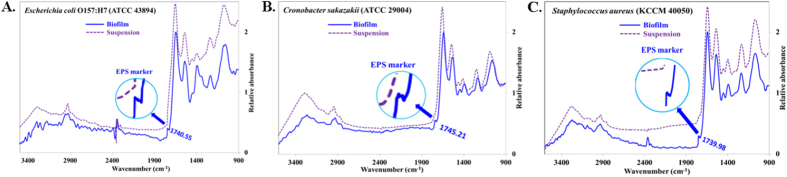
ATR-FTIR spectra of (**A**) *Escherichia coli* O157:H7 (ATCC 43894), (**B**) *Cronobacter sakazakii* (ATCC 29004), and (**C**) *Staphylococcus aureus* (KCCM 40050) biofilm and the planktonic state. Inset: A magnified view of the peak observed at 1740.55 cm^−1^.

**Figure 7:**
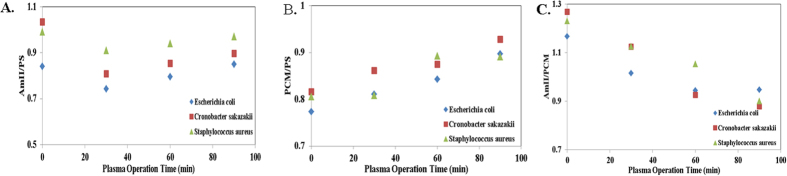
Plot of the intensity ratio of significant peak positions in comparison with the plasma operation time. Key: AmII: amide II band (~1535 cm^−1^); PS: polysaccharides (~1187 and 1052 cm^−1^); and PCM: phosphate-containing compounds (~1188 cm^−1^).

ATR-FTIR spectroscopy is a useful method for monitoring biofilms *in situ* in a non-destructive fashion, even under fully hydrated conditions^49^. Figure 5 shows the Germanium (Ge) ATR-FTIR spectra of *Escherichia coli* O157:H7 (ATCC 438), *Cronobacter sakazakii* (ATCC 29004), and *Staphylococcus aureus* (KCCM 40050) biofilms after they were treated with plasma at different time intervals including 0 min, 30 min, 60 min and 90 min. Band assignments were made according to the literature^50^,^51^,^52^,^53^,^54^,^55^,^56^,^57^,^58^, which are indicated in Fig. 5 and presented in Table 1. The major ATR-FTIR peaks obtained for different biofilms are in the region of ~3300–2900 cm^−1^, ~1640 cm^−1^, ~1535 cm^−1^, ~1444 cm^−1^, ~1390 cm^−1^, ~1235 cm^−1^, and ~1060 cm^−1^. The major ATR-FTIR peak shows a similar pattern, with a gradual decrease in peak intensity during the plasma treatment (Fig. 5) at different time intervals. To understand the effect of EPS, reference pathogens were analyzed by ATR-FTIR in suspension as well, and the ATR-FTIR spectra are shown in Fig. 6 for *Escherichia coli* O157:H7 (ATCC 438), *Cronobacter sakazakii* (ATCC 29004), and *Staphylococcus aureus* (KCCM 40050), respectively. Further analysis was performed by comparing the changes in the spectral intensity ratio (Fig. 7) of different biomolecular regions at a particular plasma operation time that provides more insight into the biochemical changes that occur during the plasma inactivation process. The peak intensity ratio changes for significant biomolecular regions such as the amide II band ~1535 cm^−1^ (AmII), phosphate-containing materials (PCM) bands at ~1235 cm^−1^, and for the polysaccharide (PS) band at ~1060 cm^−1^ were calculated and plotted in Fig. 7 for different plasma operation times. The amide II band is of particular interest because it is known as a good biomass marker and has relatively little overlap with the other bands^49^. AmII/PS (Fig. 7A) decreases markedly for the first 30 minutes and then shows an increasing trend. The AmII/PCM (Fig. 7B) ratio shows an increasing pattern, whereas PCM/PS (Fig. 7C) shows a decreasing pattern.

## Discussion

An underwater DBD plasma treatment was performed successfully for the first time to determine the inactivation mechanism of three different food-borne pathogens, namely *Escherichia coli* O157:H7 (ATCC 438), *Cronobacter sakazakii* (ATCC 29004), and *Staphylococcus aureus* (KCCM 40050), with an analytical approach using ATR-FTIR. The SE images (Fig. 5) show that the biofilms are not uniform throughout the coupon, and the pathogenic cells are covered with EPS to produce multilayered film, which increases the stability of the biofilm on the SS coupon surface. As we know, the plasma radicals have a very low level of penetration depth^27^, and therefore the radicals start disrupting the surfaces of the biofilms, which consist of an EPS layer, and then disruption continues towards the inward direction^45^. Hence, more time is required to remove the biofilm from the SS coupon in comparison with the same population level of bacterial suspensions. In our previous work, we observed that the *Escherichia coli* O157:H7 suspension (~8.0 log) was completely inactivated within two and half minutes of underwater DBD plasma treatment^10^. However, once the EPS layers were disrupted and washed away from the SS coupon by water flow, then the next layer of biofilm was exposed to the plasma radicals and disrupted.

To understand the ATR-FTIR spectra clearly, some fundamental knowledge of the cell surface characteristics and cell composition of bacteria is a prerequisite. The cell surface characteristics vary between gram-positive and gram-negative bacteria. The gram-positive bacteria have a thicker and more rigid layer of peptidoglycan (PG, 40–80% by weight of the cell wall) than gram-negative bacteria (10% by weight of the cell wall). The primary structure consists of parallel polysaccharide chains of alternating *N*-acetylglucosamine (NAG) and *N*-acetylmuramic acid (NAM) residues joined by β(1 → 4) glycosidic bonds. Variations in the protein content and teichoic acid in the peptidoglycan cell wall is observed in gram-positive and gram-negative bacteria^34^. Gram-negative bacteria have an outer membrane (OM) outside of the PG layer, which contains phospholipids in the inner layer and lipopolysaccharides in the outer layer^59^. Therefore, lower inactivation was observed in Fig. 2 for *Staphylococcus aureus* (KCCM 40050) in comparison with *Escherichia coli* O157:H7 (ATCC 438), and *Cronobacter sakazakii* (ATCC 29004). There are several interesting peaks that appear on an IR spectrum of bacteria, and most of them represent functional group vibrations in the primary biomolecular constituents such as proteins, fatty acids, nucleic acids, and carbohydrates. The 3000–2800 cm^−1^ spectral region is the fatty acid region (region I); 1700–1500 cm^−1^ contains the amide I and II bands of proteins and peptides (region II); 1500–1200 cm^−1^ is a mixed region of fatty acid bending vibrations, proteins, and phosphate-carrying compounds (region III); 1200–900 cm^−1^ corresponds to the absorption bands of the carbohydrates in microbial cell walls (region IV); and 900–700 cm^−1^ is the ‘fingerprint region’ that contains weak but very unique absorbance that are characteristic of specific bacteria (region V). Regions I and II are the most useful for routine bacterial identification; however, the other regions may be used to better understand minor variations in the structure and composition of the bacteria. This fingerprint region is significant for the discrimination of microorganisms at the strain level; therefore, in this study, region V is not included^60^.

The typical bacterial spectrum (Figs 5 and 6) is primarily the superposition of the ATR-FTIR fingerprints of five major biomolecules, and hence the peaks are comparatively broader in shape. The presence of a weak band (Fig. 6A, 6B, 6C, magnified view as an inset) in ATR-FTIR spectra of biofilms at ~1740 cm^−1^ may be the marker of EPS because the band at ~1740 cm^−1^ is completely absent for the bacterial suspension. The intensity of the membrane lipid band at 1444 cm^−1^ (ν_s_COO−) is another piece of evidence for the presence of EPS on the upper surface of biofilms because this membrane lipid band intensity was lower with respect to the AmII band in the biofilm spectrum (Fig. 6A, 6B, 6C) in comparison with the spectrum of the bacterial suspension. The exponential decrease in the evanescent wave with distance from the crystal surface is why there was a lower AmII band intensity in the biofilm. These intensity differences in the ATR-FTIR spectra for the biofilm and suspension might be the presence of different types of proteins in biofilm and bacterial suspensions. However, the cause for the greater intensity of the –NH stretching band at ~3200 cm^−1^ of the bacterial suspension than that of the band present in biofilm might be the presence of higher amounts of carbohydrates in EPS, given that EPS contains almost ~80% carbohydrates with ~20% DNA and Protein (Fig. 4). All the observations mentioned above are somewhat similar for the three different types of pathogens analyzed in this work.

The bands at ~2960 cm^−1^ and 2920 cm^−1^ (Fig. 5) starts decreasing with the increased plasma operation time, and they finally disappear because of the complete destruction of antisymmetric stretching in the CH_3_ and CH_2_ of fatty acids present in the membrane^49^. Rapid decreases were observed in the ATR-FTIR spectra of biofilm at ~2960 cm^−1^ and 2920 cm^−1^ after 60 minutes of plasma operation, showing that cell membrane disruption occurs, which is followed by the disruption of the EPS substances present in the upper surfaces of the biofilm. In the SEM images (Fig. 3), majority of the EPS layer is removed after 60 minutes of plasma treatment, and during this 60 minute treatment, most of the bacterial cells survived, and approximately 90% (Fig. 2) of the inactivated cells were killed during the operation between 60 to 90 minutes. From this observation, we can conclude that the EPS layers act as a protective film for the microbes against plasma radicals. The decrease in the peak intensity observed in Fig. 5 for the AmII band compared with the AmI band was less than the increase in the plasma operation and might be related to the complex nature of the AmII band (Fig. 6). Extensive research is needed to understand the radicals and/or ions that are primarily involved in breaking AmI and AmII bonds. Spectral intensity changes for other components such as polysaccharides, phospholipids, and nucleic acids are almost proportional with increasing plasma operation time (Fig. 5) for all three different types of pathogens analyzed in this study.

A spectral intensity ratio analysis (Fig. 7) provided us with a detailed inactivation mechanism such as that shown in Fig. 7A. The AmII/PS ratio decreases for the first 30 minutes of plasma operation, supporting the idea that the breakdown of the upper surface (EPS layer) occurs at the beginning of the plasma operation, and then the inactivation of the bacterial cell occurs. This result supports the explanation given in the previous paragraph, in which it was explained that approximately 90% of the biofilm was reduced because plasma operation occurs after the 60-minute treatment. The value for AmII/PS shows an increasing trend after 30 minutes of operation because the dead or injured bacterial cells are washed away from the stainless steel coupon. This inactivation is carried out underwater, and therefore the removal of some inner material occurs because of the disruption of the cell membrane, which may play a significant role in the decreasing and increasing phenomenon; more explanations in favor of this phenomenon will be given in the next couple of sentences. The increase of the AmII/PCM ratio shown in Fig. 7B explains the disruption of the cell membrane, which helps DNA and the phosphate-containing inner materials to come out and dissolve into water. In our previous work, DNA electrophoresis analysis data showed that the amount of genomic DNA decreased in bacterial cells after underwater DBD treatment^61^. These results validate the conclusion that DNA was released from cytoplasm because of the membrane damage that occurred with underwater DBD treatment. The PCM/PS decrease in Fig. 7C suggests that a low quantity of nucleic acid was present in the sample because the amount of dead cells increased with the increase in the plasma operation time.

Underwater DBD plasma used in this experiment generates OH radicals, reactive nitrogen species (RNS), ozone gas (O_3_) and hydrogen peroxide (H_2_O_2_)^10^. In our previous study, it was observed that after 6, 4 and 8 min of plasma treatment time the amount generated for OH radical was 1.81 × 10^−5^ M, dissolved ozone gas was 1.5 ppm and H_2_O_2_ was 2.5 × 10^−6^ M, respectively. The data shown for dissolved ozone and H_2_O_2_ until 8 and 10 min respectively, with a constant trend. In case of OH radical, it was observed that after 6–8 min of DBD operation, the intensity or concentration of 2-hydroxyterephthalic acid (HTA) or OH radical (1 mol of OH radical ≡ 1 mol of HTA) was decreased whereas the concentration of HTA (OH radical) was supposed to be either increased or remained constant. Decreasing phenomena of OH radical was described in detail with experimental evidence in the previous work^10^. Actually dissolved ozone, OH radical and H_2_O_2_ concentration remains constant throughout the 90 min of plasma treatment after 4 min, 8 min and 6 min of plasma generation, respectively. Undergoing of mutual reactions among the species generated might be the cause of remaining all three species constant throughout the plasma treatment. Detailed data was not included in this manuscript as there is no significant difference was observed with previous work in regards of final concentration of all the species, though the operation time was different. Among these radicals, the OH radicals play a major role in inactivation, with dissolved O_3_ gas ranking next in importance. H_2_O_2_ was found to play no role in *E. coli* O157:H7 inactivation in our study because the amount of hydrogen peroxide produced during our treatment time was very low (2.5 × 10^−6^ M) and remains constant throughout the operation^10^. The inactivation mechanism of *E. coli* O157:H7 via the OH radical may proceed through some chemical or physical process; OH radicals are considered to enhance chemical reactions and also have a damaging effect on the unsaturated fatty acid side chains of lipid bilayers in various cell membranes^62^,^63^. Micro-organisms in plasma are exposed to an intense attack by these radicals that likely generate surface abrasions; these abrasions cannot be repaired rapidly by the living cell. It has also been speculated that oxygen-based reactive species may have strong oxidative effects on the outer structures of cells^64^. The bactericidal effect of ozone is well known and has already been used for sterilization in various industries^65^,^66^. However, some researchers found that up to 28 ppm of ozone gas has no effect on *E. coli* O157:H7 inactivation when they performed a 30 min treatment with a DBD generator in room air at 60% relative humidity^67^. In our previous study it was observed that the effect of RNS was not much significant on planktonic cell of *E. coli* O157:H7 reduction for this current plasma set up, hence in this work no measurement was performed with respect to RNS.

From the above discussion, it is clear that underwater DBD plasma shows significant reduction effects on the pathogenic microbes used in this study, although the required inactivation time is quite a bit longer. The presence of EPS in the upper surface of biofilms plays a significant and negative role in the plasma inactivation of pathogenic microbes, regardless of the biochemical composition (in terms of carbohydrates, proteins and DNA) variations in different types of reference pathogens. ATR-FTIR can be an effective tool for observing the biochemical changes that occur during the plasma inactivation process. However, this analysis is not evident over the complete mechanism of biofilm destruction because of the inherent complexity of bacterial ATR-FTIR spectra, not only for spectral band overlapping but also for ATR technique features. In our future work, we are aiming to shorten the plasma operation time for the complete inactivation of biofilm by combining other physical techniques and/or using surfactant-based chemicals.

## Material and Methods

### Bacterial strains and preparation of inoculums

The bacteria used in this study were *Escherichia coli* O157:H7 (ATCC 438), *Cronobacter sakazakii* (ATCC 29004), and *Staphylococcus aureus* (KCCM 40050), were obtained from the bacterial culture collection of Seoul National University (Seoul, Korea). One colony from each reference bacterium was grown on Plate Count Agar (PCA, BD, Sparks, MD, USA) at 37 °C for 1 day and then used to inoculate tryptic soy broth (TSB, BD) at 37 °C for 15–16 h. The cultures were harvested and washed once by centrifugation at 10,000 × g for 10 min. The supernatant was decanted and the pelleted cells were re-suspended in phosphate-buffered saline (PBS; pH 7.4) to yield a population of approx. 4 log CFU/ml.

### Biofilm formation of bacterial strains on stainless steel coupons

The cell suspension (2 ml) was deposited in a 24-well cell culture plate (207 ml; Nasco, Fort Atkinson, WI, USA) containing a sterile stainless steel coupon (1 cm × 1 cm) and incubated at 4 °C for 24 h to facilitate cell attachment. The coupons were removed from the suspension with sterile forceps and washed in 500 ml of sterile distilled water by gently moving them in a circular motion for 5 s. The washed coupons were separately deposited in a 24-well cell culture plate containing 2 ml of TSB and incubated at 25 °C for up to 5 days to enable biofilm formation. After 1, 2, 3, 4, and 5 days, the coupons were washed in sterile distilled water (500 ml) by gently moving them in a circular motion for 5 s, and they were transferred to 10-ml conical centrifuge tubes containing 5 ml of PBS and 1 g of sterile glass beads (425–600 μm diameter; Sigma-Aldrich, 3050 Spruce Street, Saint Louis, MO 63103, USA). The mixtures were vortexed at maximum speed for 1 min. After they were vortexed, the undiluted suspensions were serially diluted in 0.85% sterile saline solution, and 100 μl of each solution was spread out on a PCA plate to incubate at 37 °C for 24 h; afterwards, the colonies were counted. The viable counts obtained for each reference strain were plotted (Fig. 1).

### Underwater electrical discharge apparatus

A schematic diagram of the experimental apparatus and conditions used in this work is shown in our previous work Khan *et al.* (AIP Adv. 2015)^10^. The underwater DBD consisted of an electrode (DBD reactor), a power supply, and a gas supply. A quartz tube with an internal diameter of 30 mm was used as a dielectric barrier. Two rod-type copper (Cu) electrodes that were 7 mm in diameter and a ground Cu electrode that was 1 mm in diameter were coiled around the quartz tube. The plasma discharge took place between the tube and the ground electrode. A neon transformer (18 kV, 20 kHz) with an input voltage of 220 V was used as the power supply. To diffuse the generated plasma, clean, dry air was fed into the inside of the quartz tube at a rate of 5 L/min, with the flow rate controlled by a regulator. The DBD reactor was submerged in an acryl-water bath (450 × 200 × 155 mm^3^), with the water in the bath acting as a coolant.

### SEM of the biofilm structures produced by bacterial strains on stainless steel coupons

Biofilms of *Escherichia coli O157:H7 (ATCC 438*)*, Cronobacter sakazakii (ATCC 29004*), and *Staphylococcus aureus (KCCM 40050*) were formed on stainless steel, and the surfaces were visualized using a scanning electron microscopy (SEM). Biofilms were formed on coupons immersed in TSB medium at 25 °C for 5 days, as described above. After biofilm formation, the coupons were rinsed in 50 ml of sterile distilled water by gently moving them in a circular motion for 5 s. The rinsed coupons were transferred to 50 ml of PBS buffer (pH 7.2) containing 0.1% concanavalin A, 0.1% CaCl_2_ and 0.1% MgCl_2_ for at least 20 min before they were rinsed with PBS (pH 7.4). The rinsed coupons were fixed in 1 M sodium cacodylate buffer containing 2.5% glutaraldehyde and 4% paraformaldehyde at 4 °C for at least 30 min and then washed three times in 0.1 M sodium cacodylate buffer for 10 min at 21 ± 2 °C. The fixed coupons were dehydrated using a graded series of ethanol concentrations (50, 60, 70, 80, and 90, and twice with 100%) for 10 min each. Coupons with dehydrated biofilms were soaked in mixtures of ethanol and isoamyl acetate at ratios of 3:1, 1:1, and 1:3 for 20 min, and finally treated twice with 100% isoamyl acetate solution for 30 min. After their treatment with the isoamyl acetate solution, the coupons were dried with 2 drops of HMDS two times for 10 min each. The coupons were coated with platinum using a Q300RT sputtering device (Quorum Technologies Ltd) for SEM analysis.

### FTIR measurements

FTIR spectra were recorded between 4,000 and 800 cm^−1^ on a Bruker Vector 22 spectrometer equipped with KBr beam splitters and deuterated triglycine sulfate (DTGS) thermal detectors. The resolution of the single beam spectra was 4 cm^−1^. FTIR measurements were performed on biofilm samples as well as on planktonic bacterial samples at 21 ± 1 °C in an air-conditioned room. Appropriate spectra were used to remove the spectral background; there was a water-reference spectrum for bacterial pellets and, for the biofilm monitoring experiments, a sample-reference spectrum was acquired immediately before the step under study. Water vapor subtraction and baseline correction were performed. The recording of the spectra, data storage and data processing were performed using Bruker OPUS 7.2.139.1294 software. A nine-reflection diamond ATR accessory (Durasampl/R^TM^, SensIR Technologies) was used to acquire spectra for bacteria with and without biofilms before and after they were treated with plasma. The incidence angle was 45° and the refraction index of the crystal was 2.4. Twenty-five scans were collected per spectrum (which corresponded to a 4-min accumulation time). For the planktonic bacterial sample in LB medium at 0.5 gL^−1^, we used a SPECAC home-modified ATR-FTIR batch cell enclosing a trapezoidal Ge crystal (72 mm × 10 mm × 6 mm, refraction index: 4.0) with an incidence angle of 45°, yielding six internal reflections on the upper face that were in contact with the sample. For biofilm monitoring, an ATR-FTIR flow cell (SPECAC) enclosing the same Ge crystal was used. The number of bidirectional double-sided interferogram scans was 100, which corresponds to a 1 min accumulation. All the interferograms were Fourier-processed using the Mertz phase correction mode and a Blackman-Harris three-term apodization function. No ATR correction was performed. The ATR spectra are shown with an absorbance scale corresponding to log (R_reference_/R_sample_), where R is the internal reflectance of the device. In ATR-FTIR mode, the sample is placed in contact with an ATR crystal. The infrared beam is focused onto the edge of the IRE, which is multiply reflected on the inner surface of the ATR crystal and then directed to a suitable detector. At each reflection at the sample-ATR crystal interface, an evanescent wave is created in the sample where it can be absorbed. The electric field amplitude of this evanescent wave decays exponentially with distance from the crystal surface. The penetration depth d_p_ of the evanescent wave is calculated by using the following equation: d_p_ = λ/[2π(n_c_^2^ sin ^2^θ − n_s_^2^)^1/2^] where λ is the wavelength of the incident radiation, n_c_ is the refractive index of the crystal, n_s_ is the refractive index of the sample in contact with the crystal, and θ is the angle of incidence. With a refractive index of the hydrated bacteria estimated at approximately 1.43 in the mid-infrared region^68^, the d_p_ was calculated to be {0.60, 1.12, and 1.58} μm, and {0.22, 0.42, and 0.59} μm at {2900, 1550 and 1100} cm^−1^ for diamond and germanium crystals, respectively. Bacteria have an average size of approximately 0.7 μm × 2–3 μm. Consequently, assuming that bacteria are in absolute contact with the ATR crystal, almost the entire bacterial cell will be analyzed with diamonds but not with Ge. Furthermore, because of the exponential decay of the electric field amplitude of the evanescent wave, the spectral contributions of different bacterial cell components depend upon their spatial localization contrary to infrared spectroscopy in transmission mode.

### Extraction of EPS

The protocol used for biochemical solution preparation was modified from a previously described method^44^. Biofilm samples (~15 ml) were thawed on ice and centrifuged at 15,000 × *g* for 20 min. The biofilm pellets were re-suspended in ~30 ml of a cold sulfuric acid solution (0.2 M sulfuric acid, pH 1.1), and the biofilm matrix was extracted into the solution as described in section 2.2. The cell suspension was stirred at 4 °C for 3 h before centrifugation at 15,000 × *g* for 20 min. The resulting supernatant was saved and used for further analysis. The biofilm dry weight was calculated by subtracting the final weight of the SS coupon after biofilm formation with the initial weight of the SS coupon, and both the weights were measured under dry conditions.

### Carbohydrate measurements

The total carbohydrate content was measured using a modified phenol-sulfuric acid method with glucose standards using total carbohydrate Assay Kit (Sigma-Aldrich, 3050 Spruce Street, Saint Louis, MO 63103, USA). Briefly, 50 μl of an EPS solution was mixed with 125 μl of concentrated sulfuric acid. Then 25 μl of 10% phenol was mixed in, and the mixture was incubated in a 95 °C water bath for 5 min. The mixture was cooled and transferred into a 96-well plate (BD Biosciences, San Jose, CA). The absorbance at 490 nm was read with a spectrophotometric plate reader (Synergy HT; BioTek, Winooski, VT) and a standard calibration method was used to measure the protein content.

### Protein measurements

To measure the total protein content, trichloroacetic acid (TCA) (final concentration, 12%) was added to the EPS solution, and the mixture was incubated on ice for 30 min before centrifugation at 15,000 × *g* for 20 min. The TCA precipitates were washed twice with 10 ml acetone and re-suspended in 2 ml of 2-*N*-morpholinoethanesulfonic acid (MES) buffer (pH 5.0). The protein content was measured using Quant-iT™ Protein Assay Kit (Invitrogen, molecular probes life technologies, Eugene, Oregon, USA). Fluorescence was measured for the mixture at 470 nm excitation and 570 nm emission maxima by using a multi-mode micro plate reader system (SpectraMax i3- Molecular Device) and a standard calibration method was used to calculate the protein content.

### DNA measurement

Quant-iT™ dsDNA Assays Kit (Invitrogen, molecular probes life technologies, Eugene, Oregon, USA) was used to the total DNA content in an EPS solution was measured after extraction with 3 volumes of 100% cold ethanol. The mixture was then incubated on ice for 2 h before DNA was recovered by centrifugation at 17,500 × *g* for 20 min at 4 °C. Fluorescence was measured for the mixture at 510 nm excitation and 527 nm emission maxima by using a multi-mode micro plate reader system (SpectraMax i3- Molecular Device) and a standard calibration method was used to calculate the DNA content.

### Statistical analysis

The data were analyzed via ANOVA using the SAS statistical program (SAS Institute, Cary, NC, USA), and the significant differences were compared using Duncan’s multiple ranges tests (*p* < 0.05). The experiments were repeated a minimum of three times unless stated otherwise.

## Additional Information

**How to cite this article**: Khan, M. S. I. *et al.* A submerged dielectric barrier discharge plasma inactivation mechanism of biofilms produced by *Escherichia coli* O157:H7, *Cronobacter sakazakii*, and *Staphylococcus aureus. Sci. Rep.* **6**, 37072; doi: 10.1038/srep37072 (2016).

**Publisher’s note:** Springer Nature remains neutral with regard to jurisdictional claims in published maps and institutional affiliations.

## Figures and Tables

**Table 1:** Assignments of principal infrared vibrational bands from the 3500–900 cm^−1^ region of the ATR-FTIR spectrum of biofilm produced by *Escherichia coli* O157:H7 (ATCC 438), *Cronobacter sakazakii* (ATCC 29004), and *Staphylococcus aureus* (KCCM 40050) on stainless steel coupons (Key: ν: stretching, δ: bending, a: antisymmetric, and s: symmetric).

Wavenumber (cm^−1^)	Assignment	Principal compounds and/or Functions	Primary corresponding cellular compounds
**3296.16**	νN-H	Amide A	Membrane
**3273.09**			
**3271.08**			
**2918.13**	ν_a_CH_3_ ν_a_CH_2_	Fatty chains	Membranes
**2927.78**			
**2929.70**			
**1739.98**	νC = O	Esters from lipids	Membranes
**1740.55**			
**1745.21**			
**1645.19**	Amide I (㯜 = O coupled with δ N–H), δ H_2_O	Proteins, water (1640 cm^−1^)	Membranes, cytoplasm, flagella, pili, ribosomes
**1637.47**			
**1531.39**	Amide II (δN–H coupled with νC–N)	Proteins	Membranes, cytoplasm, flagella, pili, ribosomes
**1535.25**			
**1537.19**			
**1444.60**	δCH_2_, δ_a_CH_3_	Lipids	Membranes
**1448.46**			
**1388.67**	ν_s_COO^−^	Amino acids, fatty acid chains	Capsule, peptidoglycan
**1394.45**			
**1398.31**			
**1234.37** **1236.30**	ν_a_PO_2_^−^	Phosphodiester, phospholipids,	Membranes, nucleoid, ribosomes
**1056.93**	ν_s_C–O–C, ν_s_P–O–C (R–O–P–O–R’)	Polysaccharides	Capsule, peptidoglycan
**1066.57**			
**1068.50**			
